# Comparative Proteomic Assessment of Normal vs. Polyhydramnios Amniotic Fluid Based on Computational Analysis

**DOI:** 10.3390/biomedicines10081821

**Published:** 2022-07-28

**Authors:** Rūta Navakauskienė, Sandra Baronaitė, Dalius Matuzevičius, Natalija Krasovskaja, Gražina Treigytė, Audronė Arlauskienė, Dalius Navakauskas

**Affiliations:** 1 Department of Molecular Cell Biology, Institute of Biochemistry, Life Sciences Center, Vilnius University, LT-10257 Vilnius, Lithuania; sandra.baronaite@santa.lt (S.B.); grazina.treigyte@gmail.com (G.T.); 2 Center for Obstetrics and Gynaecology, Clinic of Obstetrics and Gynaecology, Vilnius University Hospital Santaros Klinikos, LT-08660 Vilnius, Lithuania; 3 Electronic Systems Department, Faculty of Electronics, Vilnius Gediminas Technical University, LT-03231 Vilnius, Lithuania; dalius.matuzevicius@vilniustech.lt (D.M.); dalius.navakauskas@vilniustech.lt (D.N.); 4 Medical Genetics Center, Vilnius University Hospital Santaros Klinikos, LT-08660 Vilnius, Lithuania; natalija.krasovskaja@santa.lt; 5 Clinic of Obstetrics and Gynaecology, Faculty of Medicine, Vilnius University, LT-03104 Vilnius, Lithuania; audrone.arlauskiene@santa.lt

**Keywords:** amniotic fluid, mass spectrometry, polyhydramnios, computational analysis

## Abstract

Mass spectrometry-based proteomics have become a valued tool for conducting comprehensive analyses in amniotic fluid samples with pathologies. Our research interest is the finding and characterization of proteins related to normal vs. polyhydramnios (non-immune hydrops) pregnancy. Proteomic analysis was performed on proteins isolated from fresh amniotic fluid samples. Proteins were fractionated by 2DE using a different pI range (pI 3–11, pI 4–7) and analyzed with MALDI-TOF-MS. Furthermore, by using computational analysis, identified proteins in protein maps specific to normal vs. polyhydramnios pregnancy were compared and the quantities of expressed proteins were evaluated mathematically. Comparative analysis of proteome characteristic for the same polyhydramnios pregnancy fractionated by 2DE in different pI range (3–11 and 4–7) was performed and particular protein groups were evaluated for the quantification of changes within the same protein level. Proteins of normal and polyhydramnios pregnancies were fractionated by 2DE in pI range 3–11 and in pI range 4–7. Mass spectrometry analysis of proteins has revealed that the quantity changes of the main identified proteins in normal vs. polyhydramnios pregnancy could be assigned to immune response and inflammation proteins, cellular signaling and regulation proteins, metabolic proteins, etc. Specifically, we have identified and characterized proteins associated with heart function and circulatory system and proteins associated with abnormalities in prenatal medicine. The following are: serotransferrin, prothrombin, haptoglobin, transthyretin, alpha-1-antitrypsin, zinc-alpha-2-glycprotein, haptoglobin kininogen-1, hemopexin, clusterin, lumican, afamin, gelsolin. By using computational analysis, we demonstrated that some of these proteins increased a few times in pathological pregnancy. Computer assistance analysis of 2DE images suggested that, for the better isolation of the proteins’ isoforms, those levels increased/decreased in normal vs. polyhydramnios pregnancy, and the fractionation of proteins in pI rage 3–11 and 4–7 could be substantial. We analyzed and identified by MS proteins specific for normal and polyhydramnios pregnancies. Identified protein levels increased and/or modification changed in case of non-immune hydrops fetus and in cases of cardiovascular, anemia, growth restriction, and metabolic disorders. Computational analysis for proteomic characterization empower to estimate the quantitative changes of proteins specific for normal vs. polyhydramnios pregnancies.

## 1. Introduction

Since 1997, proteomic profiles of amniotic fluid (AF) have been created by various techniques [1]. Mass spectrometry-based proteomics has become a valuable tool for conducting comprehensive analyses in human fluid samples. Explanation of specific molecular and cellular mechanisms is crucial in order to understand all biological systems. Typically, the metabolite and protein composition of amniotic fluid alters during the gestation period. The successful progression of processes in pregnancy relies upon the intricate interrelationships between multiple biological molecules. Pregnancy disorders such as polyhydramnios, fetal growth restriction, preterm labour, and preeclampsia contribute significantly to maternal and perinatal mortality [2].

Non-immune hydrops fetalis (NIHF) pertains to anomalous fluid collections in soft tissues and serous cavities of the fetus. Fluid is usually present in more than one organ; fluid may collect in the abdominal cavity, pericardial cavity, lungs, and subcutaneous tissues [3]. NIHF is a condition easily diagnosed by an ultrasound. It includes cardiac defects, pulmonary, chromosomal abnormalities, skeletal dysplasia, fetus-maternal hemorrhage and hematologic abnormalities [4,5]. Bellini et al. stated that cardiac and liver failure, lymphatic disorders, and volume overload resulted in high central venous pressure, low oncotic pressure and reduced lymphatic flow, due to which NIHF occurred. Due to the changes in capillary permeability, the amino acid may leak into the amniotic fluid [6].

Tissues of the fetus act as endocrine organs that provide an extensive variety of peptides and proteins which get into the maternal and fetal circulation and affect several systems in various ways [7]. AF is heterogeneous in composition and, in the first half of pregnancy, the composition of AF is the same as maternal plasma except for a much lower concentration of protein [8]. As it is known, multidimensional proteomic analysis of the AF is mostly performed to recognize pathways causing the preterm birth. AF is studied for α-fetoprotein and choline-esterase to relieve the identification of open-tube defects, for definite digestive enzymes to diagnose cystic fibrosis, and for preeclampsia surfactant phospholipids to estimate the maturity of fetus lung when preterm delivery occurs. The amount of proteins like alpha-fetoprotein (AFP) in maternal serum is greatly linked to pregnancy. AFP is intensively studied because an alteration in its concentration in maternal serum is connected with the number of fetal abnormalities, including spina bifida and polyhydramnios. These proteins are generated and secreted by either the placenta or the fetus as pregnancy advances. Consequently, the expression of such markers in AF has been actively investigated for pathologies such as intra-amniotic infection, Down syndrome, trisomy’s 13 and 18, rupture of membrane, preterm delivery, preeclampsia, fetoplacental hypoxia, etc. [9,10].

In this study, we identified proteins specific for normal and polyhydramnios pregnancies. By using computational methods, we have determined their changes and excluded main proteins, the expression of which increased in normal vs. pathological pregnancies. Still, the confirmation of proteins that could be important for the prediction and diagnosis of polyhydramnios would require an extension of the study with a larger number of samples.

## 2. Materials and Methods

### 2.1. Sample Preparation

Proteomic analysis of fresh amniotic fluid samples was performed. Amniotic fluid of normal pregnancy (AFN), gestation of age 39 weeks, amniotic fluid of polyhydramnios pregnancy (AFP), and non-immune fetal hydrocephalus at 28 weeks of gestation were chosen, and the samples were prepared by amniocentesis. From both samples, proteins were isolated as described earlier [11], fractionated by 2DE and analyzed by MALDI-TOF-MS.

### 2.2. Gel Electrophoresis

The proteins were determined by two-dimensional gel electrophoresis (IEF/SDS, 2DE). An Immobiline DryStrip Kit, pH ranges 3–11 and 4–7, and Excel Gel SDS, gradient 8–18% was applied for two-dimensional electrophoresis. It was implemented based on the manufacturer’s instructions (Immobiline DryStrip Kit for 2D electrophoresis with Immobiline DryStrip and ExelGel SDS, Pharmacia Biotech, Uppsala, Sweden). To visualize the proteins, 2DE gels were stained with Colloidal Coomassie Blue-250.

### 2.3. In-Gel Digestion and MALDI-TOF MS Analysis

The relevant areas were cut off from the gel and exposed to in-gel tryptic digestion for one day. For the short term, the gel pieces were dehydrated with 50% acetonitrile and then dried entirely in a centrifugal evaporator (DNA Mini, Eppendorf). The protein spot was rehydrated in 30 μL of 25 mM ammonium bicarbonate (pH 8.3) including 20–30 μg/mL modified trypsin (Promega), and the specimens were incubated overnight at 37 °C. The tryptic peptides were then extracted from the sliced gel pieces in the following manner. Some extraneous solution left after the digestion was withdrawn and put into a fresh tube. The gel pieces were washed with 5% trifluoracetic acid in 50% acetonitrile two times, shaking from time to time. The extract and digestion solutions were afterwards merged and evaporated until the solution has dried completely. For MALDI-TOF analysis, peptides were dissolved in 3 μL of 30% acetonitrile and 0.01% trifluoracetic acid and were later set up with a matrix (alpha-cyano-4-hydroxicinnamic acid) on the target plate. The analysis was performed on a Voyager MALDI-TOF MS (Perspective Biosystems Inc., MA, USA) and externally calibrated applying synthetic peptides with known masses. Spectra were acquired in the positive ionization mode at 25 kV.

Processed data were analyzed using trypsin as the cleavage protease, one missed cleavage was allowed, and a fixed modification was set to carbamidomethylation of cysteines. The minimum identification criteria included two peptides per protein. The false discovery rate (FDR) for peptide and protein identification was determined based on the search of a reversed database, which was automatically generated using a software tool when the global false discovery rate was set to 1%. UniProtKB/SwissProt databases were used for protein identification. The identified proteins were submitted to AgBase, Version 2.0 (https://agbase.arizona.edu, last accessed on 13 September 2021) for the proteins’ function annotation.

### 2.4. Gel Image Computational Analysis

The gels were digitized with ImageScanner™ III scanner (GE Healthcare Biosciences, Laupheim, Germany) by applying LabScan 6.0 software application in particular created to obtain images from 2D electrophoresis. Scanner settings were 16-bit pixel depth and 300 dpi resolution. Images were exported as Tagged Image File Format (TIFF™) graphic files. Beforehand, the scanner was calibrated by applying the provided step tablet with known optical values. In this study, the prototype of a new software tool for 2DE gel image analysis was applied. The prototype is executed in Matlab™ environment (The MathWorks, Inc., Natick, MA, USA) and has integral original 2DE gel image processing algorithms as tools for specific tasks:

- Image preparation tools: image cropping (to remove excess areas), spot labelling, master gel selection (to align the group of gels), molecular mass markers’ calibration (to delineate area of MM marker and input of MM values), pI calibration (to input positions of known pI values);
- Image preprocessing related tools: image smoothing (to eliminate impulse noise), background elimination (to remove variations of background staining), individual image warping (to straighten protein migration paths);
- Image segmentation tools: 2DE image splitting (to split image into primary segments), segmented area evaluation (to highlight uncertain segmentations of protein spots for the user), editing of segments (to manually edit protein spots in order to remove false negatives and positives of segmentation by merging, splitting, adding, or removing areas);
- Image alignment tools: initial registration (to automatically detect some high confident control points for initial image registration), spot pairing and concluding image alignment (to find correspondences between spots), manual editing of alignment vectors (to remove mismatches and add new matches between images);
- Quantitative analysis tools: spot quantification (to measure normalized quantities of spots), changes evaluation (to calculate change ratios);
- Visualization tools: 3D viewer (to display small area of image as surface), image fusion (to display overlay of two images using pseudocolors).

The analysis of 2DE images using the listed tools is presented in the Results section. In the following, we focus on the main mathematical tools and algorithms used in image processing.

### 2.5. Image Preprocessing

The aim of preprocessing is to eliminate accidentally arising white and black pixels (impulse noise), take out background alterations, and straighten great image distortions in the paths of identical molecular mass and identical pI proteins. Image smoothing is required in order to eliminate the impulse noise that damages the images. Impulse noise occurs during image obtaining when the scanning device catches dust, small particles of stains, and other small artefacts. This type of noise can be efficiently declined by smoothing images with a local median filter that eliminates small noise elements on an image though bigger features like spots stay unaffected. The median filter replaces the centre pixel of region by the median of surrounding pixels (neighborhood).

Original background subtraction is used to remove meaningless alterations in the gel background intensity level. Gradual change of background is discovered by applying the morphological approach. We use Top-Hat operation—we subtract the morphologically opened image from the initial image. An employed structuring element is a disk.

Selection of the appropriate master gel is an important task as all the other gels will be registered (aligned) onto it. After gel image registration, all the 2DEG images will embody such absolute geometric distortions as the master gel. The major absolute geometric distortions are image distortions in the paths of identical molecular mass proteins. On the sides of 2DE gels, polypeptides noticeably tend to migrate differently (some times more, some times less) from other places. Such consequences occur because of the drawbacks of electrophoresis technology and clearly need to be compensated to ensure that polypeptides of identical molecular weight have the same horizontal position. After evaluation of the vertical geometric distortions, 2DEG images with minimal ones are selected as a master gel. The algorithm for automatic selection of the master gel consists of the following steps: (a) filtration by asymmetric median filter; (b) horizontal smoothing; (c) contour isolation by modified Canny detector; (d) removal of short contour segments; (e) polynomial approximation of contours; (f) assessment of localized distortion effects; (g) filtration of tilt coefficients; (h) evaluation of image geometric distortions; and (i) selection of the 2DEG image with minimal vertical geometric distortions. If there is no acceptable 2DEG image to be selected as a master gel, such a gel can be constructed by minimizing vertical geometric distortions. Developed software automatically identifies horizontal patterns in gels and submits likely locations for the polypeptides of identical molecular weight [12,13]. Submitted positions can be reviewed and altered by the user manually. Afterwards, the transformation function is created and applied in order to warp the image and reduce the targeted geometrical distortions.

### 2.6. Gel Image Alignment

To compare protein spots from various 2DE gel images, protein spots must be matched. Overall, during the matching procedure, the landmarks common to both images are determined. In this way, landmarks are reference points applied to warp gel images. We projected and applied a registration algorithm, which at the beginning finds several landmarks that have high certainty [13,14], carries out rigid deformation of the image based on them [15], then locates all spot correspondences between images, and eventually calculates Thin-Plate Spline Transformation [16] for overlaid visualization of images and for the pairing of the protein spots.

During the primary alignment of 2DEG images, matches in 2DEG images with the greatest reliability are searched. The alignment process consists of identification of image regions of interest, assessment of similarity of various areas of gels, and determination of matches and error search. The method to discover similar 2DEG image regions is relying on Multi-Layer Perceptron combined with comparisons of Lowe descriptors [17]. Eventually, when there is an aim to detect all spot correspondences between images, an analogous strategy is applied just limiting the search area to around the spot. After automatic spot pairing is performed, it is possible to start manual editing of alignment vectors to verify and repair mismatches and add new matches between images if required.

#### Protein Spot Detection and Segmentation

The objective of protein spot detection is to locate possible positions of protein spots. Segmentation leads to a spot boundary which delineates spot area from the background and other spots. Segmented spot area is applied as a region of interest (ROI) for computing of spot volume.

Used protein spot detection and segmentation algorithm relies on Watershed transformation together with symmetrical feature detection [16]. Initially, the rotational feature strength map *S* of preprocessed 2DE image *I* is calculated. This computation is done in calculating the second-order symmetries using the Johansson method [18]. Then, due to the complexity of calculated rotational feature strength map, only the real part of it, i.e., SR=ReS is taken and Watershed transform [19] on inverted $S_{R}$ image is applied: $S^{•(w)}=\mathrm{WST}\left(-S_{R}\right)$. Watershed transform divides image into w regions each having one maximum. The maximum indicates location of $x_{\max}^{•(w)}$ and $y_{\max}^{•(w)}$ the centre of rotational symmetry $S_{\max}^{•(w)}=\max\left(S^{•(w)}\right)$ and, at the same time—strength of it. Thus, it is the most probable location of the spot core.

Rotational symmetry gives an identical value for bright and dark spots. Thus, the second derivative is calculated on the image aiming to reject areas with bright spots. This derivative presents information on the curvature of the function. Dark protein spots arise as pits in 3D image representation; therefore, the second derivative is positive in the spot core area. This criterion is often applied in spot segmentation algorithms [13,20,21]. Due to the fact that the noise is amplified during the derivation, smoothing is used beforehand. For smoothing, a Gaussian kernel is used. The convolution property determines the application of smoothing and derivation by convolving image with derivative of Gaussian. Therefore, Laplacian of the Gaussian kernel is applied. Smoothed image regions with negative value in their centre, i.e., $I_{\mathrm{LoG}}\left(x_{\max}^{•(w)},y_{\max}^{•(w)}\right)<0$ are removed (that stipulates the elimination of a large part of the irrelevant image area). For the rest of the image regions, subsequent characteristics are extracted:

1. Mode of each region of the 2DE image;
2. Weighted difference of maximum and minimum value in contour pixels of each region of the 2DE image;
3. Weighted difference of maximum and minimum value in contour pixels of each region of the symmetry map;
4. Maximum value of Kovesi phase symmetry feature detector [22] in each region of the 2DE image;
5. Maximum Laplacian value of Gaussian in each region of the 2DE image.

According to these features, the image regions with labels were sorted into three classes: C1—“one spot exists in the region”, C2— “only part of spot exists in the region”, and C3—“there is no spot in the region” [14]. Classification was done by a Feedforward Multilayer Neural Network of three layers with 5–10–3–2 neurons and tangent sigmoid transfer functions.

Image areas sorted as having a partial spot are highlighted calling the attention of the user to review these areas and to repair (split, merge, or remove) the segmentation results interactively. Spot boundaries are determined for each gel individually, and they will be applied to delineate the area of the spot to compute the volume. After pairing and segmentation of the protein spots in gel images, the ratio of spots normalized quantities among the image groups can be computed to evaluate alterations of spot abundance between gel groups.

## 3. Results

### 3.1. Image Preparation and Preprocessing

After the images were imported to the 2D gel analysis prototype, the first step of image preparation phase was to eliminate the unneeded and incorrect parts of the image, mainly the edges of the image that are empty from the protein spots. This reduces the computational load, decreases the probability of incorrect findings, and enhances the quality of the input images prior to segmentation. In the image studied, the user interactively marks two areas—protein map analysis area and a working area that also includes MW markers. Users also can perform spot labelling, molecular mass marker calibration (to delineate area of MM marker and input of MM values), and pI calibration (input positions of known pI values).

### 3.2. Gel Image Alignment and Segmentation

When the process of automatic gel segmentation with watershed transformation has finished, the region was edited (splitted or merged) manually. Image areas that were highlighted as “containing partial spot” were studied carefully. A 3D tool was beneficial for complex region analysis. Gel image alignment was conducted as described above. After the alignment process, the isoelectric points and molecular weights of the marked protein spots were calculated.

### 3.3. Quantitative Analysis

**Figure 1:**
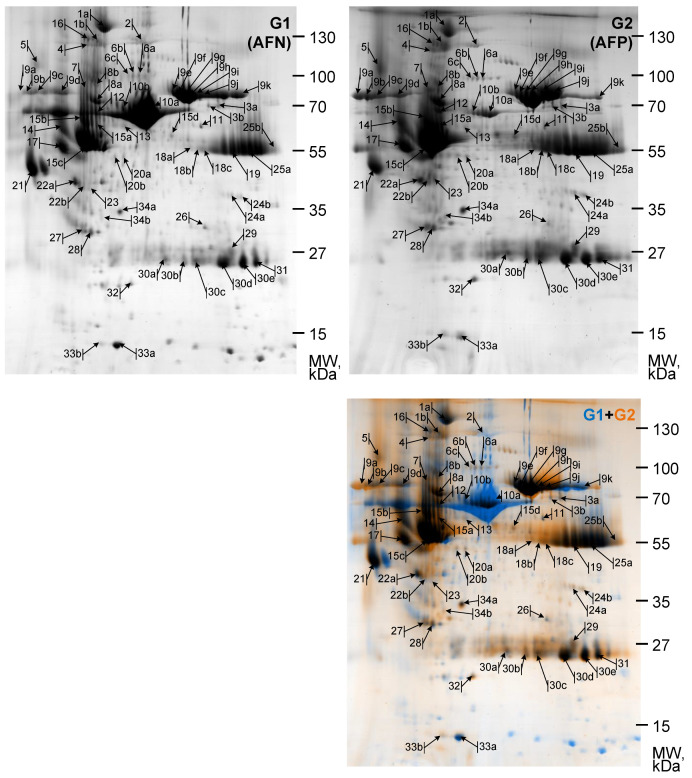
Comparative analysis of 2DE proteome maps characteristic for normal and polyhydramnios pregnancies. The proteins were resolved by 2DE, pH range 3–11, and Excel Gel SDS, gradient 8–18%. 2DE images of proteins of amniotic fluid of normal pregnancy (AFN, G1; blue in G1 + G2 and amniotic fluid of polyhydramnios pregnancy (AFP, G2; orange in G1 + G2) were superposed and presented in G1 + G2. Arrows and numbers in the 2DE maps indicate the positions of proteins supplied to MALDI-TOF MS/MS and identified. Spot labels are the same as in Table 1. Molecular weight (Mw) markers are presented on the left. Representative images from one of three experiments showing similar results are shown.

After the determination of the spot area, the number of spots is computed. Spot quantity is determined as the total intensity of a spot in a gel image and relates to the quantity of protein in the actual spot in the gel. The total intensity of an object is the sum of the intensities of all the pixels from which the object is composed. The quantity of each protein spot in a gel is divided by the total quantity of all the protein spots in that gel, in order to acquire the final normalized quantity of the given spot. The process of normalization compensates for no expression-related alterations in spot intensity. After calculating all the paired protein spots between gel images applying the integral spot quantitation tool, the ratio of spot number between images of gels G1 (representing proteins of amniotic fluid from normal pregnancy, AFN) and G2 (representing proteins of amniotic fluid from polyhydramnios pregnancy, AFP) is calculated (Figure 1, Table 1).

The normalized quantity ratio of the protein spot is applied to determine whether a spot in the first image was bigger than the corresponding spot in the second image or vice versa. The calculated values for studied protein spots are presented in Table 1 and Table 2. Because of the existence of variances in biological and technical part of the experiment, protein spots with a change of quantity larger than 2 fold are expected as a changed expression (increased or decreased).

### 3.4. Proteomic Profile Characteristics for Normal and Polyhydramnios Amniotic Fluid

The protein profiles of amniotic fluid from normal pregnancy (AFN, G1) and from polyhydramnios pregnancy (AFP, G2) fractionated in range pI 3–11 (Figure 1) were visualized, identified (Table 1) and compared (Table 1, Fold change). We analyzed approximately 100 proteins, and only 34 proteins characteristic for AFN and AFP (pI 3–11) with a proper score were successfully identified and are shown in Table 1. Most of the detected proteins are increased in the amniotic fluid of polyhydramnios pregnancy (Table 1, Fold change). Ceruloplasmin, Plasma protease C1 inhibitor, Inter-alpha-trypsin inhibitor heavy chain, Prothrombin, Alpha-1B-glycoprotein, Lumican, Basement membrane-specific heparan sulfate proteoglycan core protein, Transthyretin, and others, the protein spot level of which increased more than 2.5 times can be mentioned. The level of other proteins, e.g., like Serotransferrin in normal pregnancies vs. polyhydramnios, is changed regarding the protein spot pI, i.e., its level could depend on the protein modifications.

**Figure 2:**
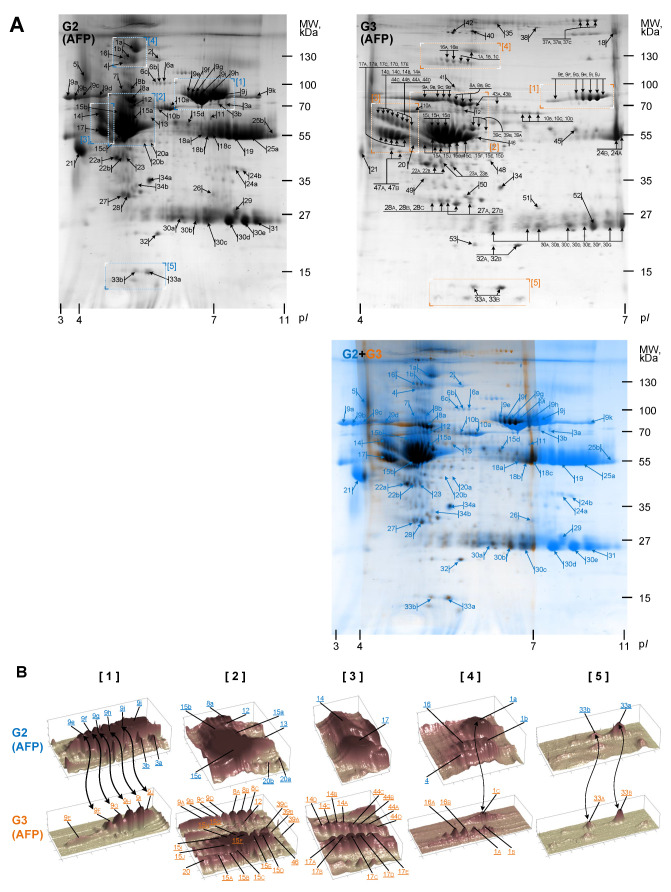
Comparative analysis of 2DE protein maps corresponding amniotic fluid of polyhydramnios pregnancy fractionated in different pI range. (**A**) proteins corresponding amniotic fluid of polyhydramnios pregnancy (AFP), fractionated in different pI range: pI 3–11 (G2, blue in G2 + G3) and pI 4–7 (G3, orange in G2 + G3) range and Excel Gel SDS, gradient 8–18%. Arrows and numbers in the 2DE maps indicate the positions of proteins supplied to MALDI-TOF MS/MS and identified. Spot labels for the proteins fractionated in the range pI 3–11 are the same as in Table 2. Spot labels for the proteins fractionated in the range pI 4–7 are the same as in Table 2. (**B**) Computational analysis of several protein groups is performed to evaluate their distribution in different pI value ranges. It shows that the same protein level with different pI changes because of modification level. In Table 2, the proteins’ spot distribution corresponding to the different modification level is presented (column— Share, %). Representative images from one of three experiments showing similar results are shown.

Individually, the characteristic proteins for AFP were separated in 2DE with a range of pI 4–7 and approximately 146 proteins were supplied for mass spectrometry analysis. Table 2 presents identified 53 proteins with high score and detailed specification. Furthermore, the images of the protein map images specific for AFP (pI 3–11 and pI 4–7) (Figure 2A(G2,G3)) were analyzed and protein spots in 2DE maps representing pI 3–11 and pI 4–7 were compared by computer assisted methods (Figure 2A(G2,G3)). Computer assisted analysis of protein spot groups’ (Figure 2B) demonstrates that some proteins like Ceruloplasmin (Figure 2B, No. 1), Alpha-1B-glycoprotein (Figure 2B, No. 8), Serotransferrin (Figure 2B, No. 9), Kininogen-1 (Figure 2B, No. 44), and others are spread in few spots with different pI when fractionated in pI 4–7. Different spots of the same protein could be associated with protein modifications. Column “Share, %” in Table 2 represents the distribution between the different points that coincide with different pI of the same protein. The changes in protein spots corresponding to different pI volumes could be valuable findings for prenatal diagnosis.

In AFP samples, we identified the higher expression of some proteins, the levels of which were higher compared to the proteome associated with normal pregnancy. The following are listed in Table 3. Their possible role for the prenatal diagnosis is discussed in the Discussion section.

**Figure 3:**
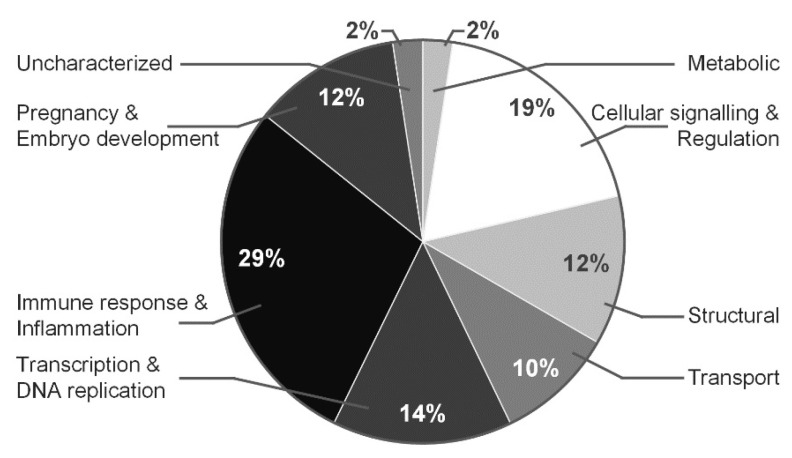
Functions of proteins identified in normal vs. polyhydramnios pregnancies, proteins fractionated in the pI 3–11 range. Proteins were clustered according to their functions by using AgBase [2.00 v].

**Figure 4:**
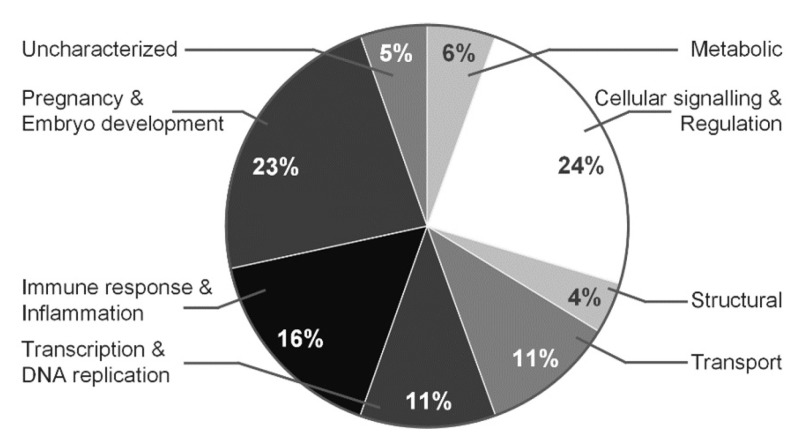
Functions of proteins identified in normal vs. polyhydramnios pregnancies, proteins fractionated in the pI 4–7 range. Proteins were clustered according to their functions by using AgBase [2.00 v].

Each identified protein was assigned to a certain group corresponding to protein function based on information from AgBase (www.agbase.msstate.edu, last accessed 13 September 2021) (Figure 3 and Figure 4). The majority of identified proteins of AFN/AFP separated in range pI 3–11 and pI 4–7 (presented in brackets) belong to immune response and inflammation proteins—29% (16%), cellular signaling and regulation proteins—19% (24%), transcription and DNA replicationproteins—14% (11%), pregnancy and embryo development proteins— 12% (23%), structural proteins—12% (4%), metabolic—2% (6%) and involved in molecular transport system—1% (11%).

## 4. Discussion

In the study, we examined protein maps characteristic for normal vs. polyhydramnios amniotic fluid. There were identified proteins, the protein levels of which increased in polyhydramnios pregnancy. The level of the number of proteins changed subject to their isoelectric points. This means that different modifications of the same protein could be important for pathological diagnosis of pregnancy. A higher concentration of proteins has been caused by pathology of polyhydramnios—non-immune hydrops fetalis (NIHF)—and it may happen due to various etiological conditions, in approximately one-third of cases, no cause could be found. Hydrops fetalis is found in about 1/2000 births [23]. One-third of hydropicfetuses are accidentally detected during prenatal ultrasound in the first or second trimester of gestation [24]. Etiologies of non-immune hydrops fetalis can be classified into groups: cardiovascular (17–35%), unknown (15–25%), lymphatic dysplasia (15.0%), chromosomal (7–16%), hematologic (4–12%), twin-twin transfusion (3–10%), infections (5–7%), thoracic (6%), syndromic (5.5%), skeletal dysplasias (3–4%), miscellaneous (3.6%), gastrointestinal (1.3%), inborn errors of metabolism (1–2%), urinary tract malformations (0.9%), and extra thoracic tumors (0.7%) [25]. Multiple cardiac lesions have been involved: the three significant subgroups are structural anomalies, vascular abnormalities, and arrhythmias. The most frequently found cardiac structural anomalies related with hydrops are atrioventricular septal defect, hypoplastic left and right heart, and isolated ventricular or atrial septal defects [23]. Evaluation of results from our study shows an increase in proteins associated with heart function and circulatory system. Results of analysis showed increased prothrombin and transthyretin relative amounts. Prothrombin as a glycoprotein occurring in blood plasma is a component of the blood-clotting mechanism. During pregnancy, alterations in the coagulation system that are seen to be adaptive to prevent hemorrhage at the time of delivery and normal pregnancy have been related with excessive maternal thrombin generation [26]. These cardiovascular proteins—prothrombin and serotransferrin—have been reported as increased in the maternal circulation in a few obstetrical syndromes as preterm labor [27,28], preeclampsia [29], and fetal growth restriction. Overall, it was demonstrated that, under oxidative stress conditions, increased phospholipase A2 activity would result in a growth of decomposition of arachidonic acid, which eventually leads to more production of prothrombin and serotransferrin [30,31]. Obstetric complications such as fetal growth, preeclampsia, rupture of membranes, restriction, preterm labor, and fetal demise is the clinical endpoint of different underlying mechanisms (i.e., infection, thrombosis, inflammation, endocrine disorder, immunologic rejection, genetic, and environmental reasons); thus, they may be considered as syndromes. Placental vascular pathology and higher thrombin generation were determined in all of these obstetrical syndromes [31,32]. A study by Karamessinis reported alterations at the post-translational modification level in alpha-2-HS glycoprotein (fetuin-A) protein in intrauterine growth restriction cases, and this glycoprotein is inextricably bound to fetal growth, cell replication, and osteogenesis [33]. It is known that transthyretin (TTR) is secreted by the liver, choroid plexus, and retinal cells, it is a homotetrameric protein which transports retinol and thyroxine (T4). The mechanism assisting in the development of hydrops in storage diseases may include obstruction of venous blood return due to organomegaly [34]. Over the past years, multiple research was conducted on TTR to resolve its participation in few amyloid diseases such as central nervous system amyloidosis (CNSA), senile systemic amyloidosis (SSA), and familial amyloid cardiomyopathy (FAC). SSA is caused by the aggregation of wild-type protein (WT-TTR), mainly in the heart, and more than 100 various point mutations are related with the other three TTR-related amyloidosis [35]. The certain pathophysiologic function of thrombin in AF still has to be identified because alterations happen despite the absence of coagulation, assuming that thrombin has other functions within the amniotic cavity that may be not associated with its task in the coagulation system [36]. Anemia provoked by hypersplenism or the decrease of erythropoietin bone marrow stem cells due to the infiltrating storage cells could be a trigger. Hypoproteinemia caused by liver dysfunction can also lead to hydrops [37,38].

As investigated, normal fetus blood plasma and hydropic fetuses show changes. Hypoalbuminemia was identified in six of the seven hydropic fetuses and in two of the non-hydropic fetuses. High albumin concentrations were also found in four hydropic fetuses, and the values of three were determined to be more than 50% of the corresponding plasma levels [39]. The findings of our study support these results.

Low weight protein clusterin (CLU) was identified in AFP (pI 3–7), which is called an enigmatic glycoprotein with apparent involvement in biological processes, and this protein was detected in diverse disease states intensifying neuronal death in hypoxia-ischemia [40]. An increased level of clusterin has been confirmed in pregnancies with pathologies in pregnancy associated with preeclampsia [41,42]. It was reported that clusterin may be the latest therapeutic target to modulate non-caspase-dependent neuronal death occurring due to acute brain injury [43]. Proteins: transthyretin (TTHY), APO-E, ceruloplasmin (CERU), α-1-microglobulin (AMBP), serum amyloid P-component (SAMP), afamin (AFAM), histidine-rich glycoprotein (HRG) and α-1-antitrypsin (A1AT), and clusterin were upregulated in AFP. All these proteins have tasks in fetal growth and development. Identified kinins are factors for the biological activity of the amniotic fluid on smooth muscle organs [44].

Ceruloplasmin was identified in an increased level in AFP; it is a copper-binding glycoprotein which has a crucial role in the metabolism and development of nervous tissue [44]. Copper participates in diverse biological reduction-oxidation processes, and is a significant cofactor of many redox enzymes. It may affect fetal lung development or pulmonary antioxidant defense. It participates in iron transport across the cell membrane and supplies Cu${}^{2+}$ ions for the ascorbate-mediated deaminase degradation of heparan sulfate chains. As it is known, ceruloplasmin levels are reduced in Wilson disease, in which copper cannot be involved into ceruloplasmin in the liver due to defects in the copper-transporting ATPase 2. Serum ceruloplasmin is the major diagnostic criteria for Wilson disease [45,46]. The alteration in the degree of copper and ceruloplasmin oxidase activity in human amniotic fluid from 20 weeks’ gestation to term was detected. The protein content of amniotic fluid reduced towards term. Ceruloplasmin demonstrated a major rise during 20–38 weeks of gestation, with a later decrease after 38 weeks [47,48]. Anagnostopoulos and co-workers reported that ceruloplasmin, alpha-1-antitrypsin, and zinc-alpha-2-glycoprotein are upregulated in specimens from pregnancies with Klinefelter syndrome fetuses, and were downregulated against proteins identified in specimens from normal fetuses [49]. Oxidative stress happens when the rate of free radical production transcends the rate of elimination by the cellular defense mechanisms. As noted earlier, oxidative stress has been related to PTL and early delivery as well as preeclampsia, PPROM, and IUGR, and other multiple conditions and diseases in the preterm infant [50].

Protein alpha-1 acid glycoprotein and complement C3 are mostly associated with metabolism (34%) and immune response (18%) [51]. Alpha-1-antitrypsin is an inhibitor of elastase and trypsin. The physiological role is the protection of the lower respiratory tract against proteolytic destruction by leukocyte elastase (HLE). For plasma protease C1, activation of the C1 complex is subordinate to the C1-inhibitor. It creates a proteolytically inactive complex with the C1r or C1s proteases. This protein can perform a task in regulating significant physiological pathways, e.g., blood coagulation, fibrinolysis, complement activation, and the generation of kinins. Alpha 1-antichymotrypsin is also related to the pathogenesis of pathology, including Alzheimer’s disease, as it strengthens the formation of amyloid-fibrils during this disease [52]. In pregnancies complicated with anencephaly and spina bifida, no substantial difference was shown for alpha-1-antichymotrypsin [53]. Alfa-2-HS-glycoprotein, also known as fetuin A, is very important in the first half of pregnancy with other proteins and is related to metabolism [54]. Glycoprotein is mostly expressed in the liver and placenta. It also piles up in bones and teeth as a major fraction of non-collagenous bone proteins. Fetuin A also plays a positive role in insulin resistance and is related to the metabolic syndrome [55].

Complement component C9 in the membrane attack complex (MAC) has a crucial role in the innate and adaptive immune response being responsible for forming pores in the plasma membrane of target cells. C9 is the pore-forming subunit of the MAC. Identified protein lumican (LUM) regulates collagen fibril organization and circumferential growth and it is a small leucine-rich proteoglycan (SLRP). Chakravarti et al. has described lumican as one of the important extracellular components in interstitial collagenous matrices of the corneal stroma, skin, aorta, lung, bone, skeletal muscle, kidney, cartilage, and intervertebral discs [56]. LUM also affects the interaction of collagen fibrils with other compounds of the extracellular matrix, being involved in its maintenance. It was identified that it interacts with fibrillar collagen [57].

Insulin-like growth factor-binding protein participates in modulating the impact of the insulin-like growth factors I and II (IGF-I and II), which play significant roles in growth, development, metabolism, and apoptosis. In early pregnancy, the IGF family (IGF peptides, IGFBPs and IGFBP proteases) has great influence on implantation and trophoblast invasion. Abnormal trophoblast invasion in early pregnancy may promote the clinic pathological entities of impaired placentation which have effects on the feto-placental unit. Impaired placentation may occur clinically in later pregnancy as preeclampsia, placental insufficiency, and intrauterine growth restriction [58,59]. The quantity of insulin-like growth factor binding protein 1 in amniotic fluid at mid pregnancy is a reliable marker of fetal growth failure [60]. In the AFP sample, we identified protein afamin whose expression is increased in cerebrovascular endothelial cells, and it eases vitamin E transport/transfer across the blood–brain barrier in a proper cell culture model [61]. This suggests the potential function of afamin in the regulation of vitamin E uptake and transport at the blood–brain barrier.

## 5. Conclusions

We observed that, in amniotic fluid with polyhydramnios, substantially different levels of several proteins are characterized. We applied computational analysis methods for proteomic characterization of amniotic fluid that empower estimating the quantitative protein changes specific to normal and polyhydramnios pregnancies. Studies show that such proteins as serotransferrin, prothrombin, kininogen-I, alpha-1-antitrypsin, zinc-alpha-2-glycoprotein, haptoglobin, lumican, and insulin-binding protein 1 could demonstrate a possible association with the pathological state of pregnancies. To confirm the importance of these molecules for the diagnostic potential and prediction of clinical risk factors, further research is needed.

## Figures and Tables

**Table 1:** The summarized search results (by UniProt, Expasy) of proteins identified from 2DE gels representing protein maps of amniotic fluid of normal and polyhydramnios pregnancies fractionated in the pI 3–11 range. An increase in spot intensity yields a positive fold-change and a decrease accordingly a negative fold-change in AFN/AFP (marked as G1/G2). **a**) AC—accession number; **b**) Score—protein Score C.I. %; **c**) Match—Matching (sequence coverage, %); **d**) TP—Theoretical Peptides; **e**) DP—Digest Peptides; **f**) FC—Fold Change.

Nmb.	AC ${}^{a)}$	Entry	Protein Description/Name	Score ${}^{b)}$	Match ${}^{c)}$	TP ${}^{d)}$	DP ${}^{e)}$	Theoretical		Experimental		FC ${}^{f)}$
								Mw, kDa	pI	Mw, kDa	pI	G1/G2
1a	P00450	CERU_HUMAN	Ceruloplasmin OS = Homo sapiens GN = CP PE = 1 SV = 1	100	20	107	21	122.128	5.44	155.47	5.00	−1.57
1b				100	12		13			137.48	4.93	−2.11
2	P01024	CO3_HUMAN	Complement C3 OS = Homo sapiens GN = C3 PE = 1 SV = 2	99.95	11	196	21	187.030	6.02	138.40	5.40	−1.05
3a	P01024	CO3_HUMAN	Complement C3 OS = Homo sapiens GN = C3 PE = 1 SV = 2	100	15	196	29	187.030	6.02	77.14	6.51	1.29
3b				98.66	14		28			76.93	6.38	1.05
4	Q14624	ITIH4_HUMAN	Inter-alpha-trypsin inhibitor heavy chain H4 OS = Homo sapiens GN = ITIH4 PE = 1 SV = 4	100	18	98	18	103.293	6.51	132.41	4.83	−5.11
5	P05155	IC1_HUMAN	Plasma protease C1 inhibitor OS = Homo sapiens GN = SERPING1 PE = 1 SV = 2	100	7	44	3	55.119	6.09	110.98	4.16	−2.93
6a	P06396	GELS_HUMAN	Gelsolin OS = Homo sapiens GN = GSN PE = 1 SV = 1	100	13	88	11	85.644	5.9	100.95	5.49	1.08
6b				94.55	2		2			100.68	5.41	−1.12
6c				100	8		7			100.47	5.35	1.12
7	P00734	THRB_HUMAN	Prothrombin OS = Homo sapiens GN = F2 PE = 1 SV = 2	99.99	5	74	4	69.992	5.64	89.00	4.80	−12.51
8a	P04217	A1BG_HUMAN	Alpha-1B-glycoprotein OS = Homo sapiens GN = A1BG PE = 1 SV = 4	100	16	45	7	54.220	5.56	79.91	4.95	−2.36
8b				99.89	11		5			92.19	4.93	−4.39
9a	P02787	TRFE_HUMAN	Serotransferrin OS = Homo sapiens GN = TF PE = 1 SV = 3	100	22	86	19	77.014	6.81	85.76	3.89	−3.49
9b				100	13		11			85.99	4.00	−1.85
9c				99.99	17		15			86.26	4.13	−2.53
9d				99.99	20		17			85.52	4.42	−3.20
9e				100	19		16			86.63	5.88	2.28
9f				100	23		20			85.03	5.94	3.04
9g				100	14		12			82.63	6.01	2.41
9h				100	15		13			82.25	6.11	1.59
9i				100	23		20			83.99	6.25	1.11
9j				100	5		4			84.30	6.39	−1.01
9k				100	19		16			84.56	7.03	2.99
10a	P02768	ALBU_HUMAN	Serum albumin OS = Homo sapiens GN = ALB PE = 1 SV = 2	100	24	88	21	69.321	5.92	70.29	5.59	5.45
10b				100	19		17			75.28	5.38	−1.37
11	P02768	ALBU_HUMAN	Serum albumin OS = Homo sapiens GN = ALB PE = 1 SV = 2	100	15	88	13	69.321	5.92	64.25	6.26	1.09
12	P02748	CO9_HUMAN	Complement component C9 OS = Homo sapiens GN = C9 PE = 1 SV = 2	100	17	70	12	63.133	5.43	72.20	4.94	−3.02
13	P01876	IGHA1_HUMAN	Ig alpha-1 chain C region OS = Homo sapiens GN = IGHA1 PE = 1 SV = 2	99.96	23	26	6	37.631	6.08	64.70	5.26	−1.32
14	P01011	AACT_HUMAN	Alpha-1-antichymotrypsin OS = Homo sapiens GN = SERPINA3 PE = 1 SV = 2	100	26	43	11	47.621	5.33	63.68	4.43	−2.11
15a	P01009	A1AT_HUMAN	Alpha-1-antitrypsin OS = Homo sapiens GN = SERPINA1 PE = 1 SV = 3	100	46	41	19	46.707	5.37	65.21	4.97	−2.11
15b				100	54		22			68.91	4.74	−4.52
15c				100	17		7			55.64	4.82	−3.24
15d				94.67	29		12			59.76	5.88	2.12
16	P01009	A1AT_HUMAN	Alpha-1-antitrypsin OS = Homo sapiens GN = SERPINA1 PE = 1 SV = 3	100	39	41	16	46.707	5.37	139.43	4.82	−5.12
17	P02765	FETUA_HUMAN	Alpha-2-HS-glycoprotein OS = Homo sapiens GN = AHSG PE = 1 SV = 1	94.42	21	29	6	39.300	5.43	54.46	4.47	−2.18
18a	P01859	IGHG2_HUMAN	Ig gamma-2 chain C region OS = Homo sapiens GN = IGHG2 PE = 1 SV = 2	99.49	19	32	6	35.878	7.66	54.11	6.12	2.05
18b				99.31	16		5			53.51	6.22	1.45
18c				99.49	19		6			53.38	6.30	1.19
19	P01860	IGHG3_HUMAN	Ig gamma-3 chain C region OS = Homo sapiens GN = IGHG3 PE = 1 SV = 2	99.5	3	40	1	41.260	8.23	52.99	6.67	−1.08
20a	P51884	LUM_HUMAN	Lumican OS = Homo sapiens GN = LUM PE = 1 SV = 2	100	23	35	8	38.405	6.16	50.27	5.26	−1.80
20b				100	17		6			50.64	5.16	−2.26
21	P02763	A1AG1_HUMAN	Alpha-1-acid glycoprotein 1 OS = Homo sapiens GN = ORM1 PE = 1 SV = 1	100	13	23	3	23.497	4.93	46.02	4.10	−1.39
22a	P25311	ZA2G_HUMAN	Zinc-alpha-2-glycoprotein OS = Homo sapiens GN = AZGP1 PE = 1 SV = 2	100	41	34	14	34.237	5.71	41.51	4.68	−4.38
22b				98.89	32		11			39.71	4.79	−4.48
23	P00738	HPT_HUMAN	Haptoglobin OS = Homo sapiens GN = HP PE = 1 SV = 1	45.47	18	45	8	45.177	6.13	39.41	4.86	−3.54
24a	P01857	IGHG1_HUMAN	Ig gamma-1 chain C region OS = Homo sapiens GN = IGHG1 PE = 1 SV = 1	99.98	18	34	6	36.083	8.46	37.54	6.67	1.86
24b				34.45	9		3			37.01	6.80	−1.63
25a	P01857	IGHG1_HUMAN	Ig gamma-1 chain C region OS = Homo sapiens GN = IGHG1 PE = 1 SV = 1	100	24	34	8	36.083	8.46	53.65	6.93	1.14
25b				100	21		7			53.23	7.30	−1.13
26	P0C0L5	CO4B_HUMAN	Complement C4-B OS = Homo sapiens GN = C4B PE = 1 SV = 2	99.36	6	187	12	192.631	6.89	29.70	6.29	1.25
27	P08833	IBP1_HUMAN	Insulin-like growth factor-binding protein 1 OS = Homo sapiens GN = IGFBP1 PE = 1 SV = 1	99.97	24	21	5	27.885	5.11	28.94	4.80	−3.41
28	P02760	AMBP_HUMAN	Protein AMBP OS = Homo sapiens GN = AMBP PE = 1 SV = 1	100	8	38	3	38.974	5.95	28.51	4.89	−1.41
29	A0M8Q6	LAC7_HUMAN	Ig lambda-7 chain C region OS = Homo sapiens GN = IGLC7 PE = 1 SV = 2	99.45	25	12	3	11.296	8.49	25.21	6.67	1.07
30a	P01834	IGKC_HUMAN	Ig kappa chain C region OS = Homo sapiens GN = IGKC PE = 1 SV = 1	54.8	27	11	3	11.602	5.58	23.89	5.77	3.89
30b				54.65	27		3			23.47	6.03	2.58
30c				96.48	18		2			23.32	6.18	1.71
30d				99.66	27		3			23.30	6.56	1.27
30e				99.93	18		2			23.43	6.81	1.33
31	P01622	KV304_HUMAN	Ig kappa chain V-III region Ti OS = Homo sapiens PE = 1 SV = 1	54.65	20	10	2	11.781	8.72	23.39	7.02	−1.38
32	P98160	PGBM_HUMAN	Basement membrane-specific heparan sulfate proteoglycan core protein OS = Homo sapiens GN = HSPG2 PE = 1 SV = 4	100	2	329	8	468.532	6.06	20.25	5.37	−2.76
33a	P02766	TTHY_HUMAN	Transthyretin OS = Homo sapiens GN = TTR PE = 1 SV = 1	100	21	14	3	15.877	5.52	14.10	5.21	−2.08
33b				47.6	36		5			14.22	5.00	−2.48
34a	P02766	TTHY_HUMAN	Transthyretin OS = Homo sapiens GN = TTR PE = 1 SV = 1	100	57	14	8	15.877	5.52	32.99	5.24	−1.82
34b				43.3	36		5			31.48	5.01	−2.61

**Table 2:** The summarized search results (by UniProt, Expasy) of proteins identified from 2DE gels representing protein maps of amniotic fluid of polyhydramnios pregnancy fractionated in pI 3–11 and pI 4–7 range. **a**) AC—accession number; **b**) Score—protein Score C.I. %; **c**) Match—Matching (sequence coverage, %); **d**) TP—Theoretical Peptides; **e**) DP—Digest Peptides; **f**) FC—Fold Change.

Nmb.	AC ${}^{a)}$	Entry	Protein Description/Name	Score ${}^{b)}$	Match ${}^{c)}$	TP ${}^{d)}$	DP ${}^{e)}$	Theoretical		Experimental		FC ${}^{f)}$
								Mw, kDa	pI	Mw, kDa	pI	G1/G2
1A	P00450	CERU_HUMAN	Ceruloplasmin OS = Homo sapiens GN = CP PE = 1 SV = 1	100	7	107	6	122.128	5.44	125.413	5.11	13.8
1B				38.76	13		11			124.394	5.2	15.9
1C				100	7		6			160.438	5.22	70.3
8A	P04217	A1BG_HUMAN	Alpha-1B-glycoprotein OS = Homo sapiens GN = A1BG PE = 1 SV = 4	95.3	20	45	7	54.220	5.56	78.913	5.1	30.7
8B				100	31		11			73.612	5.19	23.6
8C				91.35	20		8			73.460	5.22	45.7
9A	P02787	TRFE_HUMAN	Serotransferrin OS = Homo sapiens GN = TF PE = 1 SV = 3	100	24	86	17	77.014	6.81	82.460	4.72	27.6
9B				99.99	21		16			82.617	4.85	5.0
9C				99.99	17		13			82.309	4.97	7.2
9D				100	20		13			82.416	5.04	13.1
9E				92.46	15		11			81.032	6.1	12.3
9F				100	16		12			87.691	6.33	5.1
9G				100	23		17			86.703	6.37	7.7
9H				100	25		17			86.995	6.46	7.5
9I				100	24		16			87.640	6.51	6.4
9J				99.99	22		16			88.003	6.6	8.0
10A	P02768	ALBU_HUMAN	Serum albumin OS = Homo sapiens GN = ALB PE = 1 SV = 2	100	26	88	17	69.321	5.92	68.406	4.63	82.4
10B				99.81	15		10			66.982	5.83	4.6
10C				99.75	19		13			66.903	5.87	8.3
10D				97.61	24		14			66.783	5.96	4.7
12	P02748	CO9_HUMAN	Complement component C9 OS = Homo sapiens GN = C9 PE = 1 SV = 2	99.64	21	70	12	63.133	5.43	67.943	5.18	
14A	P01011	AACT_HUMAN	Alpha-1-antichymotrypsin OS = Homo sapiens GN = SERPINA3 PE = 1 SV = 2	99.99	36	43	12	47.621	5.33	63.391	4.42	28.4
14B				100	37		13			64.130	4.4	21.1
14C				100	40		14			64.980	4.37	31.8
14D				100	36		12			65.103	4.32	18.7
15A	P01009	A1AT_HUMAN	Alpha-1-antitrypsin OS = Homo sapiens GN = SERPINA1 PE = 1 SV = 3	95.46	25	41	12	46.707	5.37	52.167	4.81	2.8
15B				100	36		15			52.340	4.9	5.3
15C				99.48	34		13			51.942	4.98	1.9
15D				100	43		20			55.594	5.12	16.9
15E				100	43		18			56.410	5.06	10.0
15F				100	16		8			57.418	5.04	16.4
15G				100	45		16			58.708	5.02	19.8
15H				100	51		21			57.694	4.99	10.5
15I				100	53		22			58.746	4.93	8.3
15J				100	38		15			54.340	4.87	8.0
16A	P01009	A1AT_HUMAN	Alpha-1-antitrypsin OS = Homo sapiens GN = SERPINA1 PE = 1 SV = 3	99.99	35	41	15	46.707	5.37	125.779	4.98	65.1
16B				77.76	36	41	14			124.943	5.05	34.9
17A	P02765	FETUA_HUMAN	Alpha-2-HS-glycoprotein OS = Homo sapiens GN = AHSG PE = 1 SV = 1	99.4	15	29	4	39.300	5.43	57.132	4.31	28.2
17B				99.99	13		3			57.942	4.39	19.9
17C				98.94	14		4			57.136	4.43	18.9
17D				99.33	15		4			57.461	4.51	15.8
17E				100	15		4			56.984	4.58	17.2
18	P01859	IGHG2_HUMAN	Ig gamma-2 chain C region OS = Homo sapiens GN = IGHG2 PE = 1 SV = 2	99.86	14	32	5	35.878	7.66	246.031	6.62	
20	P51884	LUM_HUMAN	Lumican OS = Homo sapiens GN = LUM PE = 1 SV = 2	99.99	15	35	6	38.405	6.16	51.234	4.69	
21	P02763	A1AG1_HUMAN	Alpha-1-acid glycoprotein 1 OS = Homo sapiens GN = ORM1 PE = 1 SV = 1	97.2	17	23	4	23.497	4.93	48.113	4.16	
22A	P25311	ZA2G_HUMAN	Zinc-alpha-2-glycoprotein OS = Homo sapiens GN = AZGP1 PE = 1 SV = 2	100	35	34	12	34.237	5.71	42.866	4.92	40.5
22B				99.99	26		9			42.745	5.02	59.5
23A	P00738	HPT_HUMAN	Haptoglobin OS = Homo sapiens GN = HP PE = 1 SV = 1	95.86	18	45	8	45.177	6.13	44.988	4.97	68.2
23B				95.86	18	45	8			43.946	5.11	31.8
24A	P01857	IGHG1_HUMAN	Ig gamma-1 chain C region OS = Homo sapiens GN = IGHG1 PE = 1 SV = 1	100	12	34	3	36.083	8.46	58.164	6.82	33.7
24B				100	41		10			57.942	6.75	66.3
27A	P08833	IBP1_HUMAN	Insulin-like growth factor-binding protein 1 OS = Homo sapiens GN = IGFBP1 PE = 1 SV = 1	75.3	29	21	6	27.885	5.11	31.024	5.07	55.4
27B				24.73	19		4			30.460	5.3	44.6
28A	P02760	AMBP_HUMAN	Protein AMBP OS = Homo sapiens GN = AMBP PE = 1 SV = 1	98.5	8	38	3	38.974	5.95	30.648	4.9	22.4
28B				98.06	11		4			30.712	5.02	46.0
28C				100	21		8			29.843	5.17	31.5
30A	P01834	IGKC_HUMAN	Ig kappa chain C region OS = Homo sapiens GN = IGKC PE = 1 SV = 1	76.74	18	11	2	11.602	5.58	24.106	5.59	18.3
30B				92.81	18		2			25.013	5.9	6.9
30C				73.4	45		5			25.647	6.01	7.1
30D				99.97	36		4			25.687	6.32	8.2
30E				95.96	36		4			25.946	6.49	4.5
30F				54.8	27		3			26.768	6.7	24.8
30G				85.98	18		2			26.416	6.9	30.3
32A	P98160	PGBM_HUMAN	Basement membrane-specific heparan sulfate proteoglycan core protein OS = Homo sapiens GN = HSPG2 PE = 1 SV = 4	97.93	3	329	9	468.532	6.06	21.035	5.59	57.8
32B				100	5		15			21.546	5.82	42.2
33A	P02766	TTHY_HUMAN	Transthyretin OS = Homo sapiens GN = TTR PE = 1 SV = 1	100	36	14	5	15.877	5.52	13.645	5.38	51.0
33B				100	50		7			13.681	5.68	49.0
34	P02766	TTHY_HUMAN	Transthyretin OS = Homo sapiens GN = TTR PE = 1 SV = 1	100	36	14	5	15.877	5.52	34.100	5.63	
35	P02751	FINC_HUMAN	Fibronectin OS = Homo sapiens GN = FN1 PE = 1 SV = 4	34.38	16	204	28	262.460	5.46	281.640	5.43	
36	P00450	CERU_HUMAN	Ceruloplasmin OS = Homo sapiens GN = CP PE = 1 SV = 1	98.8	19	107	16	122.128	5.44	301.394	5.17	
37A	P02787	TRFE_HUMAN	Serotransferrin OS = Homo sapiens GN = TF PE = 1 SV = 3	99.95	17	86	12	77.014	6.81	282.942	6.31	35.9
37B				99.95	17		12			279.480	6.38	32.5
37C				99.99	17		7			280.342	6.44	31.5
38	P02768	ALBU_HUMAN	Serum albumin OS = Homo sapiens GN = ALB PE = 1 SV = 2	89.83	24	88	14	69.321	5.92	250.992	5.88	
39A	P01008	ANT3_HUMAN	Antithrombin-III OS = Homo sapiens GN = SERPINC1 PE = 1 SV = 1	90.29	33	60	13	52.569	6.32	58.761	5.43	18.5
39B				100	31		12			60.463	5.38	13.7
39C				100	38		16			59.465	5.34	67.8
40	P01008	ANT3_HUMAN	Antithrombin-III OS = Homo sapiens GN = SERPINC1 PE = 1 SV = 1	100	36	60	17	52.569	6.32	246.108	5.18	
41	P43652	AFAM_HUMAN	Afamin OS = Homo sapiens GN = AFM PE = 1 SV = 1	90.07	22	76	14	69.024	5.64	95.405	5.08	
42	P01009	A1AT_HUMAN	Alpha-1-antitrypsin OS = Homo sapiens GN = SERPINA1 PE = 1 SV = 3	100	17	41	7	46.707	5.37	232.691	5.03	
43A	P02790	HEMO_HUMAN	Hemopexin OS = Homo sapiens GN = HPX PE = 1 SV = 2	100	24	46	10	51.643	6.55	68.403	5.31	47.2
43B				98.19	16		6			69.730	5.52	52.8
44A	P01042	KNG1_HUMAN	Kininogen-1 OS = Homo sapiens GN = KNG1 PE = 1 SV = 2	100	15	73	10	71.912	6.34	60.418	4.62	24.2
44B				100	16		9			61.470	4.57	28.5
44C				100	13		8			63.011	4.51	36.8
44D				100	9		6			59.137	4.64	10.5
45	P02749	APOH_HUMAN	Beta-2-glycoprotein 1 OS = Homo sapiens GN = APOH PE = 1 SV = 3	19.26	8	41	3	38.273	8.34	60.134	6.33	
46	P01019	ANGT_HUMAN	Angiotensinogen OS = Homo sapiens GN = AGT PE = 1 SV = 1	71.35	22	39	7	53.121	5.87	55.993	5.37	
47A	P02750	A2GL_HUMAN	Leucine-rich alpha-2-glycoprotein OS = Homo sapiens GN = LRG1 PE = 1 SV = 2	99.85	13	32	4	38.154	6.45	50.640	4.41	56.3
47B				99.85	13		4			48.940	4.52	43.7
48	P63261	ACTG_HUMAN	Actin. cytoplasmic 2 OS = Homo sapiens GN = ACTG1 PE = 1 SV = 1	81.07	17	38	5	41.766	5.31	44.097	5.32	
49	P10909	CLUS_HUMAN	Clusterin OS = Homo sapiens GN = CLU PE = 1 SV = 1	76.74	7	59	4	52.461	5.89	37.061	4.78	
50	P02452	CO1A1_HUMAN	Collagen alpha-1(I) chain OS = Homo sapiens GN = COL1A1 PE = 1 SV = 5	99.7	3	129	4	138.857	5.6	32.106	5.25	
51	P08123	CO1A2_HUMAN	Collagen alpha-2(I) chain OS = Homo sapiens GN = COL1A2 PE = 1 SV = 7	99.98	5	122	6	129.235	9.08	29.514	6.02	
52	P18136	KV313_HUMAN	Ig kappa chain V-III region HIC OS = Homo sapiens PE = 2 SV = 2	91.75	20	10	2	14.080	6.18	26.543	6.57	
53	P02753	RET4_HUMAN	Retinol-binding protein 4 OS = Homo sapiens GN = RBP4 PE = 1 SV = 3	49.11	22	27	6	22.995	5.76	21.842	5.37	

**Table 3:** Identified AFP proteins—those expressions are higher in comparison to proteome associated with normal pregnancy in 2DE gels with pI 3–11 ranges (right panel) and AFP proteins, the spots number of which (proportionate to modification) in AFP pI 4–7 differ in comparison to fractionated in pI 3–11 range (left panel).

Proteins Corresponding Different Spots in AFP (pI 4–7)	Proteins with Higher Expression Level in AFP (pI 3–11)
Serotransferrin	Prothrombin
Kininogen-1	Alpha-1-antitrypsin
Alpha-antichymotrypsin	Inter-alpha-trypsin inhibitor heavy chain H4
Insulin-like growth factor-binding protein 1	Ig kappa chain C region
Zinc-alpha-2-glycoprotein	Zinc-alpha-2-glycoprotein
Protein AMBP	Alpha-1B-glycoprotein
Ceruloplasmin	Serotransferrin
Transthyretin	Insulin-like growth factor-binding protein 1
Haptoglobin	Complement component C9
Alpha-1-antitrypsin	Plasma protease C1 inhibitor
Kininogen	Protein AMBP
Antithrombin-III	Transthyretin
	Alpha-1B-glycoprotein
	Ceruloplasmin
	Alpha-1-antichymotrypsin
	Basement membrane-specific heparan sulfate proteoglycan core protein Alpha-1B-glycoprotein

## Data Availability

Not applicable.
